# Machine Learning Predicts Unplanned Care Escalations for Post-Anesthesia Care Unit Patients during the Perioperative Period: A Single-Center Retrospective Study

**DOI:** 10.1007/s10916-024-02085-9

**Published:** 2024-07-23

**Authors:** Andrew B. Barker, Ryan L. Melvin, Ryan C. Godwin, David Benz, Brant M. Wagener

**Affiliations:** 1 Division of Critical Care Medicine, Department of Anesthesiology and Perioperative Medicine, University of Alabama at Birmingham, 901 19th Street South, PBMR 302, Birmingham, AL 35294 United States of America; 2 Department of Anesthesiology and Perioperative Medicine, University of Alabama at Birmingham, Birmingham, AL United States of America

**Keywords:** Artificial Intelligence, Patient Safety, Precision Medicine, Predictive Analytics, Risk Stratification

## Abstract

**Background:** Despite low mortality for elective procedures in the United States and developed countries, some patients have unexpected care escalations (UCE) following post-anesthesia care unit (PACU) discharge. Studies indicate patient risk factors for UCE, but determining which factors are most important is unclear. Machine learning (ML) can predict clinical events. We hypothesized that ML could predict patient UCE after PACU discharge in surgical patients and identify specific risk factors. **Methods:** We conducted a single center, retrospective analysis of all patients undergoing non-cardiac surgery (elective and emergent). We collected data from pre-operative visits, intra-operative records, PACU admissions, and the rate of UCE. We trained a ML model with this data and tested the model on an independent data set to determine its efficacy. Finally, we evaluated the individual patient and clinical factors most likely to predict UCE risk. **Results:** Our study revealed that ML could predict UCE risk which was approximately 5% in both the training and testing groups. We were able to identify patient risk factors such as patient vital signs, emergent procedure, ASA Status, and non-surgical anesthesia time as significant variable. We plotted Shapley values for significant variables for each patient to help determine which of these variables had the greatest effect on UCE risk. Of note, the UCE risk factors identified frequently by ML were in alignment with anesthesiologist clinical practice and the current literature. **Conclusions:** We used ML to analyze data from a single-center, retrospective cohort of non-cardiac surgical patients, some of whom had an UCE. ML assigned risk prediction for patients to have UCE and determined perioperative factors associated with increased risk. We advocate to use ML to augment anesthesiologist clinical decision-making, help decide proper disposition from the PACU, and ensure the safest possible care of our patients.

**Supplementary Information:**

The online version contains supplementary material available at 10.1007/s10916-024-02085-9.

## Introduction

Since the advent of general anesthesia, the risk of mortality for elective procedures in the United States and other developed countries has never been lower [1, 2]. Despite improvements in perioperative medicine, a fraction of patients experience unexpected care escalations (UCE) following post-anesthesia care unit (PACU) discharge [3, 4]. UCE can be defined as the unforeseen need for higher acuity care, i.e. unanticipated transfer from a standard inpatient unit to an intermediate level unit, from a standard or intermediate level unit to an intensive care unit (ICU), or intervention by a Medical Emergency Team (MET). These UCE increase morbidity for patients, increase medical cost and length of hospital stay, and slow rehabilitation and recovery for patients undergoing elective surgery [3, 5].

There are a variety of clinical risk factors that may increase a patient’s risk of UCE. For example, having emergency surgery increases risk for ICU admission during the perioperative period [6, 7]. Furthermore, patients with risk factors for respiratory insufficiency after surgery, such as obesity, a diagnosis of asthma or chronic obstructive pulmonary disease (COPD), or insufficient paralytic reversal are more likely to have post-operative pulmonary complications [8, 9]. Finally, pre-operative co-morbidities such as poor nutrition, end-stage renal disease (ESRD), and chronic anemia are risk factors for poorer outcomes after surgery and anesthesia [9, 10]. While these studies indicate that there are important risk factors that may predict UCE after surgery and anesthesia, it may be difficult to determine how a combination of multiple factors conspire to predict occurrence of an UCE.

Machine learning (ML) is gaining traction in medicine and has been used to perform some tasks as well or better than well-trained physicians. Two of these contexts include interpretation of radiologic imaging [11, 12] and determination of cancer from pathology samples [13, 14]. Recently, ML has turned its attention to “big data” and the ability to predict clinical events before they occur [15, 16]. For example, studies indicate that ML has the ability to predict hypotension in the PACU [17], to predict the occurrence of ventilator-associated pneumonia after traumatic brain injury [18], and to detect heterogeneity of treatment effect in acute respiratory distress syndrome [19].

Therefore, we conducted a single center, retrospective study hypothesizing that a ML algorithm could detect patients at risk for UCE and provide risk factors that were important to UCE risk. We collected data from pre-operative visits, intra-operative records, and PACU admissions and determined which patients had an UCE. We trained a supervised ML model with this data and, after training, tested the ML model on a separate data set to determine efficacy and patient clinical factors that may be important in determining patient UCE risk.

## Methods

This study was conducted in accordance with the 1964 Declaration of Helsinki and the protocol was approved by The University of Alabama at Birmingham Institutional Review Board (Protocol Number—300007621). Consent was waived by the IRB. This manuscript adheres to the STROBE guidelines.

### Study Design and Clinical Parameters

We conducted a single center, retrospective analysis of all patients undergoing non-cardiac surgery at the University of Alabama at Birmingham from January 2016 to December 2019. All surgical patients were included if they had non-cardiac surgery (elective or emergent), were admitted to the PACU after surgery, and were admitted to an inpatient floor or intermediate care after PACU discharge. Patients were excluded if they had cardiac surgery, had outpatient surgery (discharged home after PACU stay), were directly admitted to an ICU, were < 18 years-old, or we could not determine whether they had an escalation event. We collected clinical parameters and data from patients in the form of labs collected before surgery, patient characteristics, intraoperative data, and data collected during their PACU admission from Cerner PowerChart and CompuRecord electronic medical records. These features were chosen because they represent pre-operative (hemoglobin, bicarbonate, etc.), intra-operative (blood loss, transfusion, etc.), or post-operative (vital signs, urine output (UOP), etc.) measures of end-organ dysfunction and/or morbidity. These data are completely listed in Table 1 and Supplemental Table 1. We defined an UCE as an increase in care from the inpatient floor to intermediate care or Surgical ICU (SICU), an increase in care from intermediate care to the SICU, or a MET response within 3 midnights of PACU discharge. We made this definition based on Major Adverse Cardiac Events (MACE) criteria that myocardial infarction risk (a major perioperative clinical outcome) decreases significantly after 3 midnights [20–22]. Therefore, after this timeframe, UCE is less likely to be secondary to perioperative events. For some patients, there was a MET response without escalation or delayed escalation. If this was the case, the entire spectrum of such events counted as a single UCE event.

### Machine Learning Algorithm

A “credit scorecard” modeling system [23] was applied as a set of pre- and post-processing steps layered on top of regularized logistic regression. This modeling methodology has exhibited some recent success in predicting COVID-19 risk from electronic health record data [24–26]. Variables employed are listed in Table 1 and Supplemental Table 1. 80% of patients were chosen randomly as a training cohort for ML algorithm “learning” how to determine factors that attributed UCE risk to a patient. Our supervised ML model was trained with examples where the correct answer (patient had an UCE or not) is known and used in the training process. After training, the remaining 20% of patients were used as a test cohort to determine how well the ML algorithm had learned. Continuous variables were binned into discrete categories and extant categorical variables were grouped using weight-of-evidence (WoE) binning via the Python package OptBinning [27, 28]. WoE transformations are appropriate for electronic health record data, which often contains missing and (potentially erroneous) extreme values – situations for which WoE is particularly well suited [29]. Post-regression, WoE values and model coefficients are used to calculate scorecard points [23]. While other machine learning models can handle continuous variables, the “credit scorecard” model we used requires binned variables to assign specific score points per bin. By binning the continuous variables, we provide a model that is not only predictive but also intuitively understandable and usable by healthcare providers in a real-world clinical setting. Additionally, WoE binning process of continuous variables also has advantages in terms of robustness and stability. Furthermore, it has the “nice” theoretical property of ensuring a monotonic relationship of predictors and outcomes. Additionally, it can mitigate the influence of outliers and less frequent or missing values which might skew the results in a model that uses continuous values without binning [26, 29]. The resulting model is straightforward to interpret and easy to implement.

Elastic-net regularized logistic regression served as both a variable selection method and hedge for colinear variables [30]. The optimal regularization strength and mixing parameter for elastic-net regression was chosen using 10-fold cross-validation. The final scorecard model range of values was enforced upon the model by solving a mixed-integer programming problem constrained by the WoE bins and results of the regularized logistic regression model. This final step enforces whole-number point values, avoiding decimals in the final scorecard – adding to the ease of interpretation.

The final total score needs to accurately reflect the patient’s risk of UCE, but for interpretability and ease of calculation, we wanted this to be a number between 0 and 100. This goal of getting a score that both reflects the risk accurately and stays within a specific range is called the objective function in a mixed integer programming nomenclature [31].

The mixed integer programming model adjusts the points assigned to each patient factor (called the decision variables) to optimize the scoring within the set constraints. The constraints themselves, such as the total score needing to be between 0 and 100, are the parameters. The key outcome of this process is a set of scorecard points for each factor, which when added together for a patient, gives a total score that predicts the risk of UCE within the desired range of 0 to 100.

Using mixed integer programming here is akin to finely tuning a balance scale. The algorithm adjusts the weights (the points for each factor) to achieve a balanced and accurate reading (the total score). This must be done so that the final scores not only remain within the desired scoring range of 0 to 100 but also as closely as possible reflect the true risk of UCE based on the underlying logistic regression model.

For those wishing to reproduce our work, a copy of the code and parameters used to produce our scorecard model is available at https://github.com/UABPeriopAI/post-pacu-uce.

### Statistical Analysis

Shapley Additive exPlainations (SHAP) is a framework built around Shapley values to explain ML models [32–34]. By iteratively removing each variable from the model and looking at the effects on all other variables, Shapley values can quantify the influence of each variable. Decision plots are examples of how Shapely values influence the estimated probability. Each line in the plot represents a patient and the color gradient goes from red to blue as highest to lowest estimated probability of UCE. The line moving further right on the plot indicates an increase in risk probability, where some lines move left across the variables to indicate a decrease in probability of UCE risk. The variables are ordered by magnitude of importance.

Continuous variables were compared using a two-sample t-test and categorical variables (including Boolean variables) were compared with Chi-squared tests or Fisher’s exact test, as applicable. Correction for multiple comparisons was performed using Hommel’s method [35], as implemented in the Python package “TableOne” [36]. A p-value of < 0.05 was considered significantly different.

## Results

**Fig. 1:**
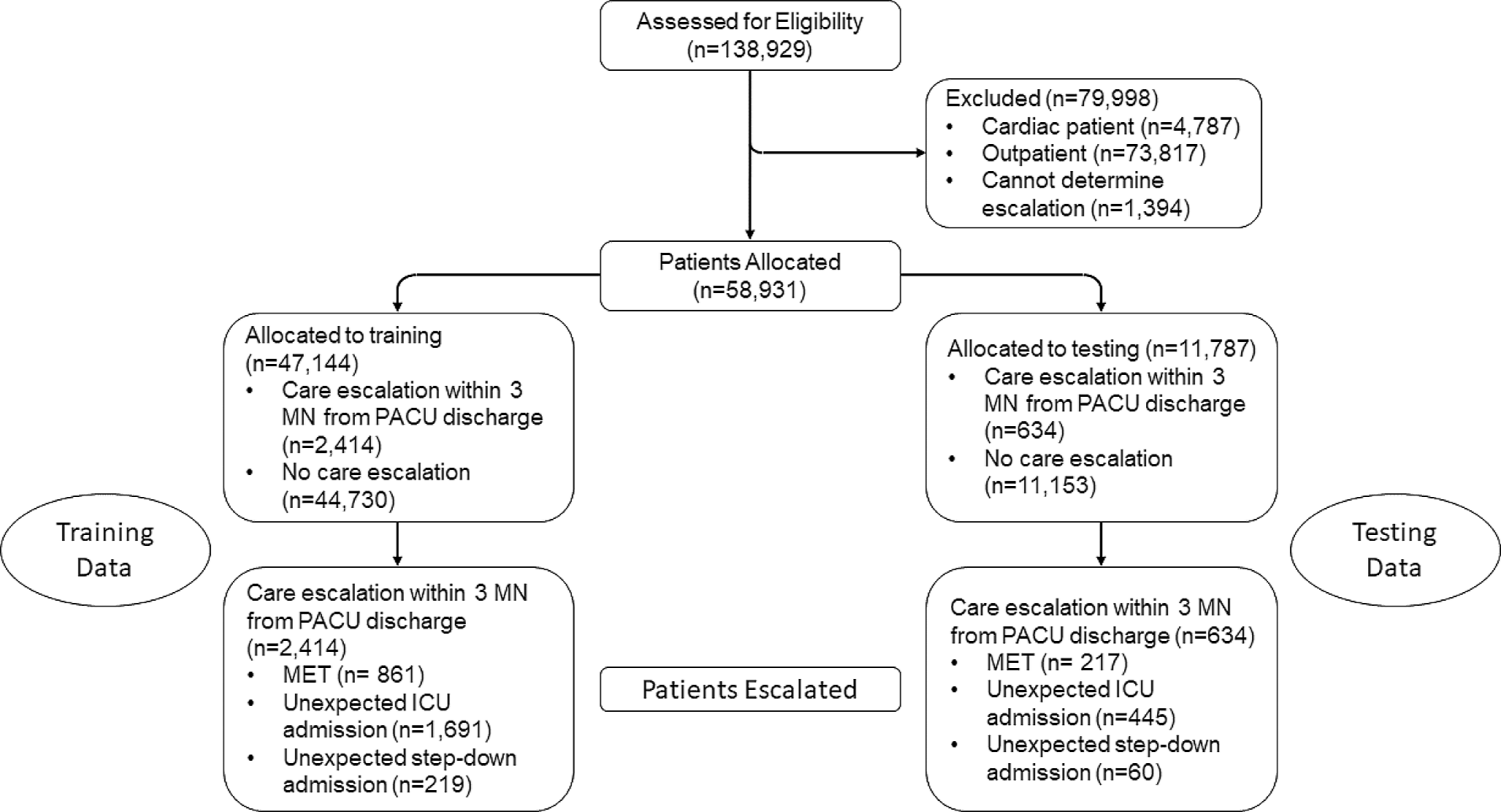
Study Design Flow Chart. The total number of patients assessed, excluded, included, and allocated to the different arms of the study are shown. Furthermore, the number of patients within in each arm that had UCE are indicated

We conducted a retrospective analysis of all surgical inpatients at the University of Alabama at Birmingham from January 2016 and December 2019. Patients were included in the study and had UCE determined as described in the *Methods*. We screened 138,929 patients during the study timeframe and 79,998 were excluded per the *Methods* (Fig. 1). A total of 58,931 patients were included in the study and 47,144 (80%) were randomly allocated to the training group and the remainder, 11,787, were allocated to the testing group. The training and testing groups had an UCE incidence rate of 5.12% and 5.37% (*p* > 0.99), respectively, indicating no significant differences. Additionally, the reason(s) for UCE (MET, ICU admission, and intermediate care admission) occurred at similar rates between the training and testing groups.

Table 1 shows patient demographics and clinical characteristics for the overall cohort (*n* = 58,931), training group (*n* = 47,144), and testing group (*n* = 11,787). For the overall cohort, 46.4% were male, mean age was 51.7 years, and mean body-mass index was 30.4. Roughly 50% of the cohort was Caucasian and 32.8% were African-American. At the time of surgery, 12.3% had chronic kidney disease and 3.8% had ESRD; 18.3% developed acute kidney injury (AKI) during the preoperative period. Before surgery, 3.3% of patients had COPD, 4.5% had asthma, and 12.5% had diabetes listed on their pre-operative problem list. The mean score for MACE was 0.9, the most prevalent ASA Status score was 3 (69.8%), and 9.3% of cases were emergencies. UCE rate was 5.2%. There were no differences in demographics or clinical characteristics between the training or testing groups. A more complete list of variables collected for the overall cohort, training group, and testing group is available (Supplemental Table 1). In both Table 1 and Supplemental Table 1, we include a column that lists the number of events that are missing from a discrete category. For example, there are no data missing for sex or age, but there are 1,353 events missing for the overall cohort for race.

**Table 1:** Patient demographics and clinical characteristics

	Missing (*n*)	Overall	Training Set	Testing Set	*p* (adjusted)
Patients; n	0	58,931	47,144	11,787	n/a
**Sex**	0				
Female; n (%)		31,514 (53.5)	25,190 (53.4)	6324 (53.7)	> 0.99^C^
Male; n (%)		27,373 (46.4)	21,913 (46.5)	5460 (46.3)	
Undetermined; n (%)		44 (0.1)	41 (0.1)	3 (0.0)	
Age; mean (SD)	0	51.7 (18.2)	51.6 (18.1)	52.0 (18.2)	> 0.99
BMI; mean (SD)	4759	30.4 (12.5)	30.4 (12.5)	30.5 (12.4)	> 0.99
**Race**	1353				
American Indian or Alaska Native; n (%)		126 (0.2)	100 (0.2)	26 (0.2)	> 0.99^C^
Asian; n (%)		648 (1.1)	528 (1.1)	120 (1.0)	
African American; n (%)		18,858 (32.8)	15,045 (32.7)	3813 (33.1)	
Hispanic or Latino; n (%)		1484 (2.6)	1206 (2.6)	278 (2.4)	
Multiple; n (%)		487 (0.8)	390 (0.8)	97 (0.8)	
Native Hawaiian/Other Pacific Islander; n (%)		6 (0.0)	6 (0.0)		
Other; n (%)		6273 (10.9)	5003 (10.9)	1270 (11.0)	
Unknown; n (%)		824 (1.4)	648 (1.4)	176 (1.5)	
Caucasian; n (%)		28,872 (50.1)	23,131 (50.2)	5741 (49.8)	
**Ethnicity**	2383				
Hispanic/Latino; n (%)		1415 (2.5)	1147 (2.5)	268 (2.4)	> 0.99^C^
Multiple; n (%)		19 (0.0)	17 (0.0)	2 (0.0)	
Non-Hispanic/Latino; n (%)		53,274 (94.2)	42,561 (94.1)	10,713 (94.5)	
Not reported; n (%)		1840 (3.3)	1488 (3.3)	352 (3.1)	
CKD; n (%)	0	7269 (12.3)	5809 (12.3)	1460 (12.4)	> 0.99^C^
AKI; n (%)	0	10,780 (18.3)	8574 (18.2)	2206 (18.7)	> 0.99^C^
ESRD; n (%)	0	2217 (3.8)	1791 (3.8)	426 (3.6)	> 0.99^C^
COPD; n (%)	0	1942 (3.3)	1567 (3.3)	375 (3.2)	> 0.99^C^
Asthma; n (%)	0	2669 (4.5)	2145 (4.5)	524 (4.4)	> 0.99^C^
Diabetes; n (%)	0	7383 (12.5)	5901 (12.5)	1482 (12.6)	> 0.99^C^
MACE; mean (SD)	35,694	0.8 (3.4)	0.8 (3.7)	0.8 (2.4)	> 0.99
Emergency; n (%)	0	5468 (9.3)	4391 (9.3)	1077 (9.1)	> 0.99^C^
**ASA Status; n (%)**	6				
1		917 (1.6)	753 (1.6)	164 (1.4)	> 0.99^C^
2		12,253 (20.8)	9867 (20.9)	2386 (20.2)	
3		41,153 (69.8)	32,816 (69.6)	8337 (70.7)	
4		4572 (7.8)	3681 (7.8)	891 (7.6)	
5		28 (0.0)	20 (0.0)	8 (0.1)	
6		2 (0.0)	2 (0.0)		
UCE; n (%)	0	3048 (5.2)	2414 (5.1)	634 (5.4)	> 0.99^C^

BMI: Body Mass Index; CKD: Chronic Kidney Disease; AKI: Acute Kidney Injury; ESRD: End Stage Renal Disease; COPD: Chronic Obstructive Pulmonary Disease; MACE: Major Adverse Cardiac Events; ASA Status is a physical status classification system to score patient fitness before surgery. Of note, in the *p*−value column, “C” denotes use of Chi−squared test and “F” denotes use of Fisher−exact test

**Fig. 2:**
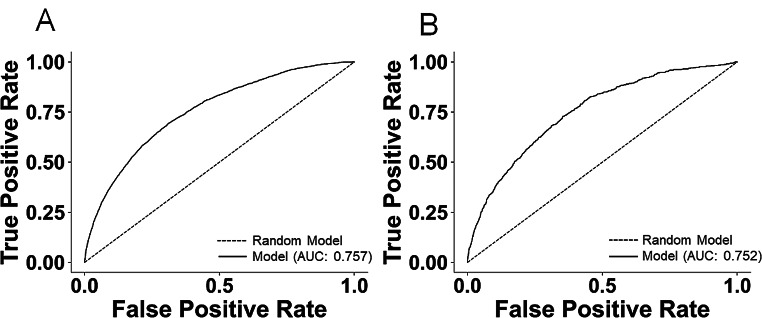
ML algorithm learns and can predict UCE in post-PACU discharge patients within three midnights. (**A**) Testing and (**B**) training receiver-operator curves are displayed. For both figures, what a random ML model would produce is shown with a dashed line. The AUC for training (**A**) is 0.757 and testing (**B**) is 0.752, both of which are acceptable values for ML

After acquiring available patient data, we trained a supervised ML model to detect differences in patients who had an UCE within 3 midnights of PACU discharge with those who did not. We used the Credit Card modeling system as it incorporates missing data and includes it as a risk factor. Using our training data, the model was able to predict UCE risk with an AUC of 0.757 (Fig. 2A). After the model had learned using 80% of patients in the study, it was applied to a test group of patients and achieved an AUC of 0.752 (Fig. 2B). These are both acceptable AUC scores with ML models [37]. Across the training and test groups, the model found similar percentages of patients within the risk bins it created, and within each of these bins, the patient’s UCE risk is not significantly different, with the exception of the second bin (Supplemental Fig. 1). These data indicate that the model functions as expected within a dataset collected from the same institution.

After model training, we examined how the model predicted risk for each patient characteristic or clinical parameter that was provided. For each datum, the model created bins for continuous variables and assigned risk with points for each bin (Table 2). For example, non-surgical anesthesia time is a risk factor for UCE and the longer this time is the higher the risk the model assigns. Additionally, the model applies appropriate risk to heart rates that are too low or too high in the last hour of PACU care. Finally, low SpO_2_ values are accorded more risk by the model.

**Table 2:** Breakdown of example bins with percentage of events within each bin and points scored by machine learning algorithm for being within a bin

Variable	Bin	Count (%)	Points
**Non-surgical Anesthesia Time**	(-inf, 30.50)	6.9	3
	[30.50, 46.50)	18.5	4
	[46.50, 53.50)	14.3	4
	[53.50, 62.50)	19	5
	[62.50, 68.50)	10.2	6
	[68.50, 80.50)	13.8	7
	[80.50, 88.50)	5.6	8
	[88.50, inf)	9.9	10
	Missing	1.9	0
**Maximum HR during last hour of PACU stay**	(-inf, 63.50)	5.8	2
	[63.50, 71.50)	9.8	0
	[71.50, 77.50)	10.3	1
	[77.50, 83.50)	11.9	1
	[83.50, 94.50)	24.2	2
	[94.50, 96.50)	5.1	3
	[96.50, 106.50)	11.4	4
	[106.50, inf)	5.8	8
	Missing	15.8	2
**Last SpO**_**2**_ **before PACU discharge**	(-inf, 94.50)	8.2	2
	[94.50, 95.50)	8	0
	[95.50, 97.50)	23.3	0
	[97.50, 98.50)	13.8	0
	[98.50, 99.50)	13.4	0
	[99.50, inf)	32.6	1
	Missing	0.6	7

HR: Heart Rate; PACU: Post−Anesthesia Care Unit

**Fig. 3:**
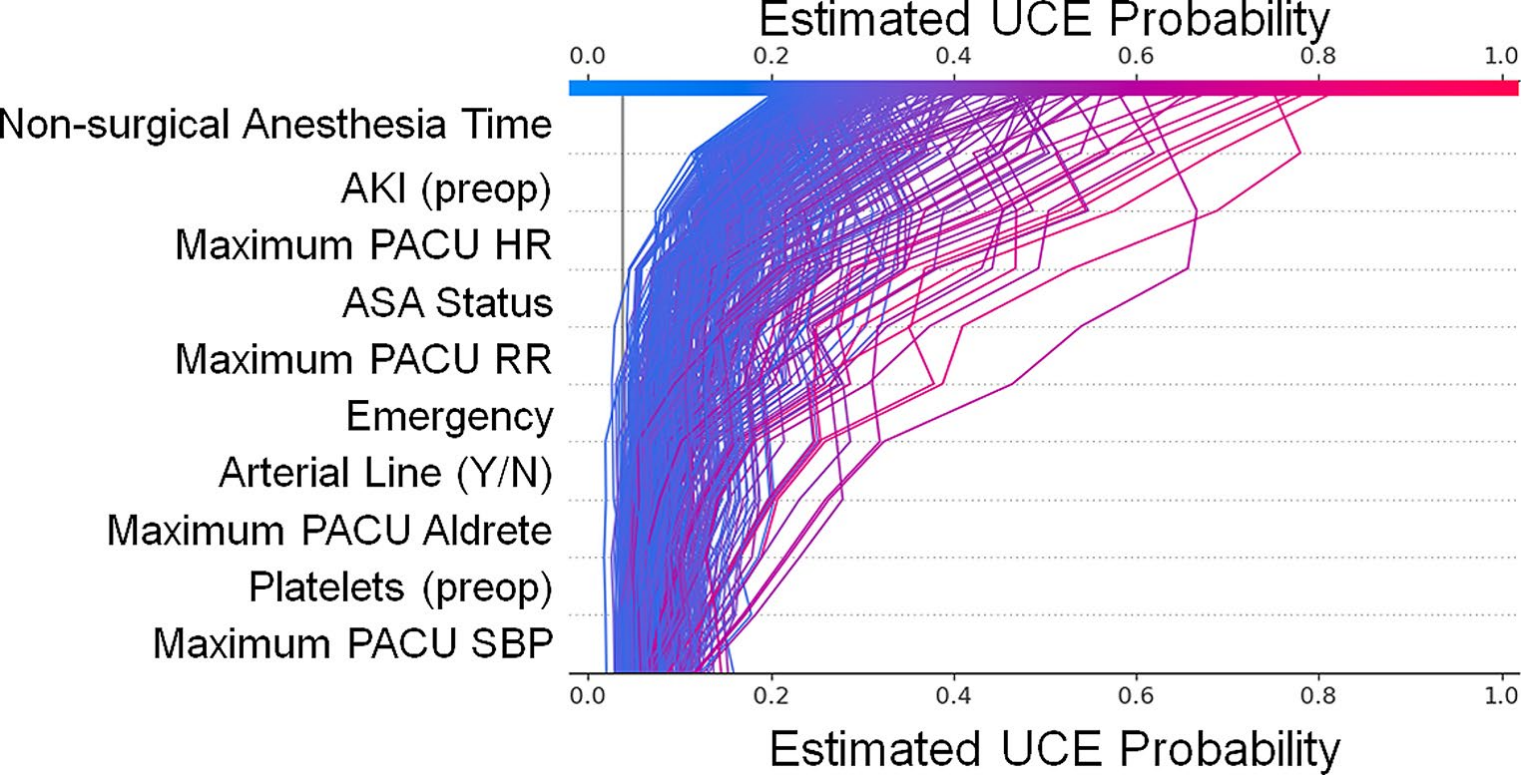
Shapley values that most affect patient UCE risk. On the left, the 10 most influential variables for patients whose estimated probability of UCE was greater than 20% are listed. Each patient is visualized as a path moving from lower risk (left-hand side) to higher risk (right-hand side) as variables of increasing importance (moving from bottom to top) are evaluated for that patient. The greater a patient moves to the right, the more risk that variable provided for that patient. If they move to the left, the variable caused a decrease in risk of UCE for that patient. Of note, any value labeled as “Maximum PACU” (e.g. HR, RR, Aldrete, SBP) is measured in the last hour of the PACU stay

We next sought to understand the variables that had the greatest effect on patient UCE risk. We found the 10 most influential variables for patients whose estimated probability of UCE was greater than 20% (Fig. 3). For each patient, their path is visualized as moving from lower risk (left-hand side) to higher risk (right-hand side) as variables of increasing importance (moving from bottom to top) are evaluated for that patient. The five most influential variables as determined by SHAP values were non-surgical anesthesia time, AKI, maximum heart rate in the last hour before PACU discharge, ASA status, and maximum respiratory rate in the last hour before PACU discharge. Complete analysis of all variables and their influence in described in Supplemental Fig. 2. To further understand which specific bins within each variable provided the highest patient UCE risk, we examined any bin variable that provided 5 or more risk points from the ML algorithm (Table 3). For reference, the maximum score of the scorecard is 100, while the minimum is 0. For a visualization of the association of a scorecard score from this model with actual risk, see Supplemental Fig. 2. There are thirteen bins that provide 5 or more risk points: non-surgical anesthesia time, SpO_2_, systolic blood pressure, heart rate, respiratory rate, emergency case, ASA status > 3, and Aldrete score. Interestingly, non-surgical anesthesia time was the most important variable identified by SHAP values. Any duration longer than 53.5 min scored 5 or more points with longer durations equating to larger point totals. A non-surgical anesthesia time duration longer than 88.5 min was the largest single contributor to the ML model with 10 points. Finally, and intriguingly, a missing value that should have been measured in the last hour before PACU discharge also plays an important role in the risk probability for an UCE. For example, a missing SpO_2_ measurement, systolic blood pressure measurement, or Aldrete score in the last hour before PACU discharge all caused the ML model to score 5 points to patient UCE risk.

**Table 3:** Individual variables that provide the highest risk points in the ML model

Variable	Bin	Count (%)	Points
**Non-surgical Anesthesia Time**	[88.50, inf)	0.1	10
Maximum PACU HR*	[106.50, inf)	0.097	8
**Non-surgical Anesthesia Time**	[80.50, 88.50)	0.055	8
**Last PACU SpO**_**2**_ *	Missing	0.14	7
**ASA Status**	[3.50, inf)	0.099	7
**Non-surgical Anesthesia Time**	[68.50, 80.50)	0.05	7
**Non-surgical Anesthesia Time**	[62.50, 68.50)	0.037	6
**Non-surgical Anesthesia Time**	Special	0	6
Maximum PACU SBP*	Missing	0.114	5
Maximum PACU RR*	[25.50, inf)	0.086	5
Minimum PACU Aldrete*	Missing	0.073	5
**Emergency (Y/N)**	[38]	0.065	5
**Non-surgical Anesthesia Time**	[53.50, 62.50)	0.03	5

* These values are measured in the final hour of the PACU stay. HR: Heart Rate; PACU: Post−Anesthesia Care Unit; ASA: American Society of Anesthesiologists; SBP: Systolic Blood Pressure; RR: Respiratory Rate

## Discussion

We hypothesized that ML could predict patients that may experience UCE and identify factors that increased UCE risk. We compared patients who experienced an UCE to those that did not to determine whether the model could find these patients and identify specific risk factors associated with UCE. Our study obtained the following significant results: (1) the rate of UCE was ~ 5%; (2) ML can be used to find patients at risk for UCE; (3) there are specific risk factors associated with UCE; and (4) after training, the algorithm identifies risk factors indicative of increased UCE risk similar to that known by anesthesiologists and the literature *without prompting*.

The UCE rate was approximately 5% in the training and testing groups. This is an increased incidence rate compared to other studies that have examined unplanned ICU admission [3]. It is generally accepted that the risk of post-operative MACE decreases three days after surgery [20–22]. We therefore used “three midnights” as our time-frame for the assessment of post-operative UCE. Furthermore, we define UCE as an incident of increased patient care level or MET response; including from the inpatient floor to intermediate care or an unplanned ICU admission. We included MET response within our definition as this typically indicates a point of clinical decline requiring medical intervention. These broader definitions are a likely reason we demonstrate an increased UCE incidence.

Numerous ML algorithms detect risk within specific patient populations or problems better than physicians. A recent example includes determining proper endotracheal tube depth [39]. Our algorithm can analyze individual patient data prior to PACU discharge and assign UCE risk levels within the following three midnights to individual patients. A primary motivation for our *a priori* approach (scorecard modeling) was to enhance the interpretability and clinical applicability of our model. While other machine learning models can handle continuous variables, the “credit scorecard” model we used requires binned variables to assign specific score points per bin. The scorecard model was selected because it can provide a straightforward, easy-to-understand risk score for clinicians. The Aldrete Score was developed to help determine when a patient is ready to be discharged from the PACU [40, 41]. While highly useful to determine readiness for discharge and recovery from anesthesia, the score is not designed to determine illness severity or proper unit disposition. A model that can analyze larger pools of data quickly and attribute UCE risk to surgical patients would detect patients at risk of clinical decompensation and augment anesthesiologist clinical decision making regarding appropriate disposition. Furthermore, these results could lead to a new scoring system that is better able to decide appropriate disposition for post-surgical PACU patients. Other published algorithms also consider patient escalation and predict when patients will do poorly [42–44]. These studies consider UCE throughout the hospital stay or consider patient events 1 year post-discharge, but are not specific to the surgical patient or the perioperative period. Future studies should focus on comparison with other warning system scores [45, 46] for greater generalizability to non-surgical hospital patients.

Our ML algorithm was able to identify specific risk factors for patient UCE (Fig. 3; Table 2). Some of these risks are already known—AKI, maximal respiratory rate, etc. However, two new variables were non-surgical anesthesia time and “missing” data. First, increased non-surgical anesthesia time incorporates both a timeframe between anesthesia start time and surgical procedure start time and another timeframe between the surgical procedure finish time and anesthesia finish time. Importantly, different events occur during each timeframe. Interestingly, increased non-surgical anesthesia time is likely due to patient characteristics, procedural technicalities, and process variables. For example, before the surgical procedure, there may be placement of an arterial line or central venous line, an unanticipated difficult airway, or difficulty in patient positioning. After the procedure, the patient could have a prolonged awakening time after anesthesia and/or co-morbidities, such as age or cardiac/respiratory diagnoses, that prolong extubation. It is currently unclear whether the patient risk factors lead to needed procedures or the procedures and other processes themselves are responsible for increased non-surgical anesthesia time. Second, “missing” data in the last hour before PACU discharge increased UCE risk. The clinical concern is that patient data is not available for patient evaluation before PACU discharge. Whether missing data is normal or abnormal and how this data would be interpreted by the ML model to attribute UCE risk is unclear. Unfortunately, patient decisions were made without this data in the past. However, in future prospective studies, we hope that because our algorithm indicates a higher UCE risk score when critical patient data is missing, a physician or PACU nurse will be alerted, the data can be added to the algorithm, and more accurate evaluations of patient UCE risk can be made. Furthermore, testing this variable prospectively will improve the algorithm and, perhaps, find other risk factors for patient UCE that can be intensely studied. Both of the above findings are sources of future investigation.

Data from the model indicate some demographic and/or clinical factors that increase UCE risk are known. This is paramount and beneficial to this study as the ML model applied scores, *without assistance*, to these variables and agree with changes in patient factors that would similarly concern anesthesiologists regarding patient clinical status. Knowing that the ML model views clinical patient factors similar to that found in clinical practice, should improve trust between anesthesiologists and AI and should be viewed as augmentation of our discipline’s clinical skillset [47–49]. For example, many of the diagnoses—emergency, increased heart rate, respiratory rate, AKI, etc.—are known to cause perioperative mortality and morbidity for patients [6–10]. However, most studies examine these factors as individual entities rather than in tandem. It would be difficult for anesthesiologists to evaluate the number of variables that the ML model evaluates for every patient discharged from the PACU at large medical centers. However, a ML algorithm can do this for every patient in the PACU quickly and alert anesthesiologists to patients that meet predetermined UCE risk levels and indicate factors causing the ML algorithm to attribute risk. Identification of patient UCE risk will augment anesthesiologist clinical practice as they can examine select patient charts for specific risk elements and make informed decisions about the most appropriate patient care levels (inpatient floor, intermediate care, or ICU). Furthermore, this will improve communication with surgical teams to alert them of patient UCE risk factors and concern for closer monitoring. In our study where ~ 5% of patients had an UCE, this became an average of ~ 600 patients/year or 2 patients/day that would require evaluation at our specific institution. The potential benefit to the patient should outweigh the cost to anesthesiologists, streamline analysis of multiple variables, and accentuate our perioperative practice.

Weaknesses of the study include inclusion of only a single center, no prospective testing of the model, and only non-cardiac surgery patients. While this is a single center study, it employs nearly 60,000 patient records and, as can be seen in Table 1, we have rich diversity among age, race, gender, and co-morbidities of patients, improving the generalizability of our results. Despite no prospective testing of the model, our model did test well after training and was able to find risk factors that are similar to that known by anesthesiologists and the literature *without prompting*. This is a positive indicator for the algorithm as it is not frequently including obsequious risk factors. Cardiac surgery patients were excluded from our study because nearly 100% of them go immediately to an ICU after surgery. Determining risk of re-entry, post-operative bleeding, etc. in cardiac surgery patients will require a different analysis and algorithm. These three limitations are the goals of future studies.

In conclusion, this ML algorithm can be used to predict patients that are at increased UCE risk. While it cannot absolutely tell anesthesiologists where to place patients within the hospital, it does provide us with increased data to predict patients that will have a problem within the next three midnights. This data will be useful as it allows us to closely evaluate these patients prior to PACU discharge, speak with their surgical teams, and provide interventions that may reduce morbidity for these patients during their hospital stay. Future studies are needed to investigate: (1) prospective use of the model; (2) testing against an external cohort; and (3) the possibility of a new scoring system to better evaluate PACU discharge and appropriate care level. These findings are exciting as they use ML to augment anesthesiologist decision-making, further our role in precision medicine and the perioperative period, and provide the safest possible care of our patients.

## Electronic Supplementary Material

Below is the link to the electronic supplementary material.

Supplementary Material 1

## Data Availability

No datasets were generated or analysed during the current study.

## Declarations

### Standards of Reporting

This manuscript adheres to the STROBE guidelines.

## References

[1] D. Bainbridge, J. Martin, M. Arango, D. Cheng, and G. Evidence-based Peri-operative Clinical Outcomes Research, “Perioperative and anaesthetic-related mortality in developed and developing countries: a systematic review and meta-analysis,” *Lancet*, vol. 380, no. 9847, pp. 1075-81, Sep 22 2012, doi: 10.1016/S0140-6736(12)60990-8. 10.1016/S0140-6736(12)60990-8 22998717

[2] D. A. Watters *et al*, “Perioperative mortality rate (POMR): a global indicator of access to safe surgery and anaesthesia,” World J Surg, vol. 39, no. 4, pp. 856 – 64, Apr 2015, doi: 10.1007/s00268-014-2638-4. 10.1007/s00268-014-2638-4 24841805

[3] N. Katori, K. Yamakawa, K. Yagi, Y. Kimura, M. Doi, and S. Uezono, “Characteristics and outcomes of unplanned intensive care unit admission after general anesthesia,” BMC Anesthesiol, vol. 22, no. 1, p. 191, Jun 20 2022, doi: 10.1186/s12871-022-01729-y. 35725372 10.1186/s12871-022-01729-y PMC9208222

[4] M. S. Melton *et al*, “Unplanned hospital admission after ambulatory surgery: a retrospective, single cohort study,” *Can J Anaesth*, vol. 68, no. 1, pp. 30–41, Jan 2021, doi: 10.1007/s12630-020-01822-1. Admission non planifiee a l’hopital apres une chirurgie ambulatoire: une etude retrospective de cohorte unique. 10.1007/s12630-020-01822-1 33058058

[5] T. J. Loftus *et al*, “Overtriage, Undertriage, and Value of Care after Major Surgery: An Automated, Explainable Deep Learning-Enabled Classification System,” J Am Coll Surg, vol. 236, no. 2, pp. 279–291, Feb 1 2023, doi: 10.1097/XCS.0000000000000471. 36648256 10.1097/XCS.0000000000000471 PMC9993068

[6] H. Ohbe, H. Matsui, R. Kumazawa, and H. Yasunaga, “Intensive care unit versus high dependency care unit admission after emergency surgery: a nationwide in-patient registry study,” Br J Anaesth, vol. 129, no. 4, pp. 527–535, Oct 2022, doi: 10.1016/j.bja.2022.06.030. 35961814 10.1016/j.bja.2022.06.030

[7] G. Costa *et al*, “Gastro-intestinal emergency surgery: Evaluation of morbidity and mortality. Protocol of a prospective, multicenter study in Italy for evaluating the burden of abdominal emergency surgery in different age groups. (The GESEMM study),” Front Surg, vol. 9, p. 927044, 2022, doi: 10.3389/fsurg.2022.927044. 36189400 10.3389/fsurg.2022.927044 PMC9524583

[8] I. Garutti *et al*, “Spontaneous recovery of neuromuscular blockade is an independent risk factor for postoperative pulmonary complications after abdominal surgery: A secondary analysis,” Eur J Anaesthesiol, vol. 37, no. 3, pp. 203–211, Mar 2020, doi: 10.1097/EJA.0000000000001128. 32028288 10.1097/EJA.0000000000001128

[9] D. Chandler *et al*, “Perioperative strategies for the reduction of postoperative pulmonary complications,” Best Pract Res Clin Anaesthesiol, vol. 34, no. 2, pp. 153–166, Jun 2020, doi: 10.1016/j.bpa.2020.04.011. 32711826 10.1016/j.bpa.2020.04.011

[10] H. Mufti *et al*, “The association between preoperative anemia, blood transfusion need, and postoperative complications in adult cardiac surgery, a single center contemporary experience,” J Cardiothorac Surg, vol. 18, no. 1, p. 10, Jan 7 2023, doi: 10.1186/s13019-023-02132-5. 36611177 10.1186/s13019-023-02132-5 PMC9824911

[11] A. Hosny, C. Parmar, J. Quackenbush, L. H. Schwartz, and H. Aerts, “Artificial intelligence in radiology,” *Nat Rev Cancer*, vol. 18, no. 8, pp. 500–510, Aug 2018, doi: 10.1038/s41568-018-0016-5. 10.1038/s41568-018-0016-5 PMC6268174 29777175

[12] J. Seah, T. Boeken, M. Sapoval, and G. S. Goh, “Prime Time for Artificial Intelligence in Interventional Radiology,” Cardiovascular and interventional radiology, vol. 45, no. 3, pp. 283–289, Mar 2022, doi: 10.1007/s00270-021-03044-4. 35031822 10.1007/s00270-021-03044-4 PMC8921296

[13] V. Baxi, R. Edwards, M. Montalto, and S. Saha, “Digital pathology and artificial intelligence in translational medicine and clinical practice,” Mod Pathol, vol. 35, no. 1, pp. 23–32, Jan 2022, doi: 10.1038/s41379-021-00919-2. 34611303 10.1038/s41379-021-00919-2 PMC8491759

[14] K. Bera, K. A. Schalper, D. L. Rimm, V. Velcheti, and A. Madabhushi, “Artificial intelligence in digital pathology - new tools for diagnosis and precision oncology,” Nat Rev Clin Oncol, vol. 16, no. 11, pp. 703–715, Nov 2019, doi: 10.1038/s41571-019-0252-y. 31399699 10.1038/s41571-019-0252-y PMC6880861

[15] S. Mainali and S. Park, “Artificial Intelligence and Big Data Science in Neurocritical Care,” Crit Care Clin, vol. 39, no. 1, pp. 235–242, Jan 2023, doi: 10.1016/j.ccc.2022.07.008. 36333034 10.1016/j.ccc.2022.07.008

[16] R. Thirunavukarasu, G. P. D. C, G. R, M. Gopikrishnan, and V. Palanisamy, “Towards computational solutions for precision medicine based big data healthcare system using deep learning models: A review,” Comput Biol Med, vol. 149, p. 106020, Oct 2022, doi: 10.1016/j.compbiomed.2022.106020. 36088715 10.1016/j.compbiomed.2022.106020

[17] K. Palla *et al*, “Intraoperative prediction of postanaesthesia care unit hypotension,” Br J Anaesth, vol. 128, no. 4, pp. 623–635, Apr 2022, doi: 10.1016/j.bja.2021.10.052. 34924175 10.1016/j.bja.2021.10.052 PMC9074793

[18] A. Abujaber, A. Fadlalla, D. Gammoh, H. Al-Thani, and A. El-Menyar, “Machine Learning Model to Predict Ventilator Associated Pneumonia in patients with Traumatic Brain Injury: The C.5 Decision Tree Approach,” Brain Inj, vol. 35, no. 9, pp. 1095–1102, Jul 29 2021, doi: 10.1080/02699052.2021.1959060. 10.1080/02699052.2021.1959060 34357830

[19] P. Sinha, A. Spicer, K. L. Delucchi, D. F. McAuley, C. S. Calfee, and M. M. Churpek, “Comparison of machine learning clustering algorithms for detecting heterogeneity of treatment effect in acute respiratory distress syndrome: A secondary analysis of three randomised controlled trials,” *EBioMedicine*, vol. 74, p. 103697, Dec 2021, doi: 10.1016/j.ebiom.2021.103697. 10.1016/j.ebiom.2021.103697 PMC8645454 34861492

[20] N. H. Badner, R. L. Knill, J. E. Brown, T. V. Novick, and A. W. Gelb, “Myocardial infarction after noncardiac surgery,” *Anesthesiology*, vol. 88, no. 3, pp. 572-8, Mar 1998, doi: 10.1097/00000542-199803000-00005. 10.1097/00000542-199803000-00005 9523798

[21] G. Landesberg *et al*, “Importance of long-duration postoperative ST-segment depression in cardiac morbidity after vascular surgery,” *Lancet*, vol. 341, no. 8847, pp. 715-9, Mar 20 1993, doi: 10.1016/0140-6736(93)90486-z. 10.1016/0140-6736(93)90486-z 8095624

[22] I. Vascular Events In Noncardiac Surgery Patients Cohort Evaluation Study *et al*, “Association between postoperative troponin levels and 30-day mortality among patients undergoing noncardiac surgery,” *JAMA*, vol. 307, no. 21, pp. 2295 – 304, Jun 6 2012, doi: 10.1001/jama.2012.5502. 10.1001/jama.2012.5502 22706835

[23] X. Dastile, Celik, T, Potsane, M, “Statistical and machine learning models in credit scoring: A systematic literature survey,” Applied Soft Computing, vol. 91, p. 106263, 2020, doi: 10.1016/j.asoc.2020.106263.

[24] L. Jehi *et al*, “Development and validation of a model for individualized prediction of hospitalization risk in 4,536 patients with COVID-19,” PLoS One, vol. 15, no. 8, p. e0237419, 2020, doi: 10.1371/journal.pone.0237419. 32780765 10.1371/journal.pone.0237419 PMC7418996

[25] L. Jehi *et al*, “Individualizing Risk Prediction for Positive Coronavirus Disease 2019 Testing: Results From 11,672 Patients,” *Chest*, vol. 158, no. 4, pp. 1364–1375, Oct 2020, doi: 10.1016/j.chest.2020.05.580. 10.1016/j.chest.2020.05.580 PMC7286244 32533957

[26] T. K. K. Mamidi, T. K. Tran-Nguyen, R. L. Melvin, and E. A. Worthey, “Development of An Individualized Risk Prediction Model for COVID-19 Using Electronic Health Record Data,” Front Big Data, vol. 4, p. 675882, 2021, doi: 10.3389/fdata.2021.675882. 34151259 10.3389/fdata.2021.675882 PMC8211871

[27] G. Navas-Palencia, “Optimal binning: mathematical programming formulation,” doi: 10.48550/arXiv.2001.08025.

[28] G. Navas-Palencia, “Optimal Counterfactual Explanations for Scorecard modelling,” doi: 10.48550/arXiv.2104.08619.

[29] L. P. Zdravevski E, Kulakov A, “Weight of evidence as a tool for attribute transformation in the preprocessing stage of supervised learning algorithms,” *The* 2011 *International Joint Conference on Neural Networks*, pp. 181–188, 2011, doi: 10.1109/IJCNN.2011.6033219.

[30] H. Zou, Hastie, T, “Regularization and variable selection via the elastic net.,” Journal of the Royal Statistical Society: Series B (Statistical Methodology), vol. 67, pp. 301–320, 2005, doi: 10.1111/j.1467-9868.2005.00503.x.

[31] M. J. Alves and J. Climaco, “A review of interactive methods for multiobjective integer and mixed-integer programming,” European Journal of Operational Research, vol. 180, no. 1, pp. 99–115, 2007.

[32] *The Shapley Value: Essays in Honor of Lloyd S. Shapley*. Cambridge: Cambridge University Press, 1998.

[33] R. Aumann, *Game Theory*. Palgrave Macmillan UK, 1989.

[34] S. Lundberg, Lee, SI, “A unified approach to interpreting model predictions,” Advances in neural information processing systems, 2017, doi: 10.48550/arXiv.1705.07874.

[35] S. Y. Chen, Z. Feng, and X. Yi, “A general introduction to adjustment for multiple comparisons,” J Thorac Dis, vol. 9, no. 6, pp. 1725–1729, Jun 2017, doi: 10.21037/jtd.2017.05.34. 28740688 10.21037/jtd.2017.05.34 PMC5506159

[36] T. J. Pollard, A. E. W. Johnson, J. D. Raffa, and R. G. Mark, “tableone: An open source Python package for producing summary statistics for research papers,” JAMIA Open, vol. 1, no. 1, pp. 26–31, Jul 2018, doi: 10.1093/jamiaopen/ooy012. 31984317 10.1093/jamiaopen/ooy012 PMC6951995

[37] J. V. Carter, J. Pan, S. N. Rai, and S. Galandiuk, “ROC-ing along: Evaluation and interpretation of receiver operating characteristic curves,” *Surgery*, vol. 159, no. 6, pp. 1638–1645, Jun 2016, doi: 10.1016/j.surg.2015.12.029. 10.1016/j.surg.2015.12.029 26962006

[38] G. Aad *et al*, “Observation of associated near-side and away-side long-range correlations in sqrt[s(NN)] = 5.02 TeV proton-lead collisions with the ATLAS detector,” *Physical review letters*, vol. 110, no. 18, p. 182302, May 3 2013. [Online]. Available: http://www.ncbi.nlm.nih.gov/pubmed/23683193. 10.1103/PhysRevLett.110.182302 23683193

[39] M. H. Huang *et al*, “Validation of a Deep Learning-based Automatic Detection Algorithm for Measurement of Endotracheal Tube-to-Carina Distance on Chest Radiographs,” *Anesthesiology*, vol. 137, no. 6, pp. 704–715, Dec 1 2022, doi: 10.1097/ALN.0000000000004378. 10.1097/ALN.0000000000004378 36129686

[40] J. A. Aldrete and D. Kroulik, “A postanesthetic recovery score,” *Anesth Analg*, vol. 49, no. 6, pp. 924 – 34, Nov-Dec 1970. [Online]. Available: https://www.ncbi.nlm.nih.gov/pubmed/5534693. 5534693

[41] D. Yamaguchi *et al*, “Usefulness of discharge standards in outpatients undergoing sedative endoscopy: a propensity score-matched study of the modified post-anesthetic discharge scoring system and the modified Aldrete score,” BMC Gastroenterol, vol. 22, no. 1, p. 445, Nov 4 2022, doi: 10.1186/s12876-022-02549-7. 36333660 10.1186/s12876-022-02549-7 PMC9635164

[42] A. Kia *et al*, “MEWS++: Enhancing the Prediction of Clinical Deterioration in Admitted Patients through a Machine Learning Model,” J Clin Med, vol. 9, no. 2, Jan 27 2020, doi: 10.3390/jcm9020343. 10.3390/jcm9020343 PMC7073544 32012659

[43] M. J. Rothman, S. I. Rothman, and J. t. Beals, “Development and validation of a continuous measure of patient condition using the Electronic Medical Record,” *J Biomed Inform*, vol. 46, no. 5, pp. 837 – 48, Oct 2013, doi: 10.1016/j.jbi.2013.06.011. 10.1016/j.jbi.2013.06.011 23831554

[44] S. Jahandideh, G. Ozavci, B. W. Sahle, A. Z. Kouzani, F. Magrabi, and T. Bucknall, “Evaluation of machine learning-based models for prediction of clinical deterioration: A systematic literature review,” Int J Med Inform, vol. 175, p. 105084, Jul 2023, doi: 10.1016/j.ijmedinf.2023.105084. 37156168 10.1016/j.ijmedinf.2023.105084

[45] Y. M. Hydoub *et al*, “Risk Prediction Models for Hospital Mortality in General Medical Patients: A Systematic Review,” Am J Med Open, vol. 10, Dec 2023, doi: 10.1016/j.ajmo.2023.100044. 10.1016/j.ajmo.2023.100044 PMC10715621 38090393

[46] M. E. Smith *et al*, “Early warning system scores for clinical deterioration in hospitalized patients: a systematic review,” Annals of the American Thoracic Society, vol. 11, no. 9, pp. 1454–65, Nov 2014, doi: 10.1513/AnnalsATS.201403-102OC. 25296111 10.1513/AnnalsATS.201403-102OC

[47] F. Pesapane *et al*, “Myths and facts about artificial intelligence: why machine- and deep-learning will not replace interventional radiologists,” *Med Oncol*, vol. 37, no. 5, p. 40, Apr 3 2020, doi: 10.1007/s12032-020-01368-8. 10.1007/s12032-020-01368-8 32246300

[48] A. Di Ieva, “AI-augmented multidisciplinary teams: hype or hope?,” *Lancet*, vol. 394, no. 10211, p. 1801, Nov 16 2019, doi: 10.1016/S0140-6736(19)32626-1. 10.1016/S0140-6736(19)32626-1 31699402

[49] O. Asan, A. E. Bayrak, and A. Choudhury, “Artificial Intelligence and Human Trust in Healthcare: Focus on Clinicians,” *J Med Internet Res*, vol. 22, no. 6, p. e15154, Jun 19 2020, doi: 10.2196/15154. 10.2196/15154 PMC7334754 32558657

