# Supplementary material for: Machine Learning Predicts Unplanned Care Escalations for Post-Anesthesia Care Unit Patients during the Perioperative Period: A Single-Center Retrospective Study
Source: J Med Syst. 2024 Jul 23;48(1):69. doi: 10.1007/s10916-024-02085-9 (PMC11266221; doi:10.1007/s10916-024-02085-9)

**Supplementary Tables, Figures, and Figure Legends**

for

**Machine Learning Predicts Unplanned Care Escalations for Post-Anesthesia Care Unit Patients During the Perioperative Period: A Single-Center Retrospective Study**

Andrew B. Barker^1,#^, Ryan L. Melvin^2,#^, Ryan C. Godwin^2^, David Benz^2^, Brant M. Wagener^1,*^

ORCID ID B. M. W.— 0000-0001-7889-1526

^1^Division of Critical Care Medicine, Department of Anesthesiology and Perioperative Medicine, University of Alabama at Birmingham, Birmingham, Alabama, United States of America

^2^Department of Anesthesiology and Perioperative Medicine, University of Alabama at Birmingham, Birmingham, Alabama, United States of America

^#^These authors contributed equally to the work

^*^Corresponding author: Brant M. Wagener, M.D., Ph.D., 901 19^th^ Street South, PBMR 302, Division of Critical Care Medicine, Department of Anesthesiology and Perioperative Medicine, Heersink School of Medicine, University of Alabama at Birmingham, Birmingham, AL 35294; Phone: (205) 934-2369; E-mail: [bwagener@uabmc.edu](mailto:bwagener@uabmc.edu)

**Supplemental Table 1. Patient Demographics and Clinical Characteristics.**

|  | **Missing (n)** | **Overall** | **Training Set** | **Testing Set** | ***p* (adjusted)** |
| --- | --- | --- | --- | --- | --- |
| Minimum PACU SpO2; mean (SD) | 220 | 97.8 (3.3) | 97.8 (3.2) | 97.7 (3.3) | >0.99 |
| Last PACU SpO2; mean (SD) | 370 | 97.8 (2.4) | 97.8 (2.3) | 97.8 (2.4) | >0.99 |
| Maximum PACU O2 Flow Rate; mean (SD) | 25583 | 1.1 (1.5) | 1.1 (1.5) | 1.1 (1.5) | >0.99 |
| Last PACU O2 Flow Rate; mean (SD) | 25963 | 1.0 (1.4) | 1.0 (1.4) | 1.0 (1.5) | >0.99 |
| Maximum PACU HR; mean (SD) | 9002 | 85.1 (14.8) | 85.1 (14.8) | 85.2 (14.8) | >0.99 |
| Minimum PACU SBP; mean (SD) | 299 | 125.6 (18.7) | 125.6 (18.6) | 125.5 (18.7) | >0.99 |
| Maximum PACU SBP; mean (SD) | 299 | 135.9 (22.8) | 136.0 (22.8) | 135.8 (23.0) | >0.99 |
| Minimum PACU MAP; mean (SD) | 2541 | 83.4 (15.5) | 83.5 (15.5) | 83.2 (15.6) | >0.99 |
| Maximum PACU MAP; mean (SD) | 2541 | 88.1 (13.1) | 88.2 (12.9) | 88.0 (14.0) | >0.99 |
| Last PACU MAP; mean (SD) | 2841 | 85.3 (13.4) | 85.4 (13.3) | 85.2 (13.5) | >0.99 |
| Maximum PACU RR; mean (SD) | 210 | 18.4 (4.2) | 18.4 (4.2) | 18.4 (4.2) | >0.99 |
| Max PACU Pain Score; mean (SD) | 5918 | 3.8 (3.3) | 3.8 (3.3) | 3.8 (3.3) | >0.99 |
| Minimum PACU Aldrete Score, mean (SD) | 2048 | 8.7 (1.0) | 8.7 (1.0) | 8.7 (1.0) | >0.99 |
| Albumin Level (preop); mean (SD) | 26986 | 3.6 (0.7) | 3.6 (0.7) | 3.6 (0.7) | >0.99 |
| Opioid Abuse; n (%) | 0 | 632 (1.1) | 503 (1.1) | 129 (1.1) | >0.99^C^ |
| Sleep Apnea; n (%) | 0 | 18 (0.0) | 11 (0.0) | 7 (0.1) | >0.99^F^ |
| Alcohol Use; n (%) | 0 | 2014 (3.4) | 1606 (3.4) | 408 (3.5) | >0.99^C^ |
| EBL; mean (SD) | 9001 | 234.0 (362.7) | 233.2 (359.2) | 237.3 (376.6) | >0.99 |
| Sugammadex (mg); mean (SD) | 0 | 41.8 (102.5) | 41.3 (102.0) | 43.8 (104.3) | >0.99 |
| Neostigmine (mg); mean (SD) | 0 | 1.2 (1.9) | 1.2 (1.8) | 1.2 (2.4) | >0.99 |
| pRBC transfused (ml); mean (SD) | 0 | 21.0 (121.2) | 20.9 (119.7) | 21.2 (126.7) | >0.99 |
| FFP transfused (ml); mean (SD) | 0 | 2.6 (46.1) | 2.4 (43.1) | 3.4 (56.7) | >0.99 |
| Cryoprecipitate transfused (ml); mean (SD) | 0 | 0.0 (2.4) | 0.0 (2.4) | 0.1 (2.7) | >0.99 |
| Platelets transfused (ml); mean (SD) | 0 | 0.8 (18.8) | 0.8 (19.3) | 0.8 (16.5) | >0.99 |
| Cell Saver transfused (ml); mean (SD) | 0 | 1.5 (29.3) | 1.3 (25.2) | 2.1 (41.8) | >0.99 |
| Normosol infused (ml); mean (SD) | 0 | 17.0 (167.9) | 16.9 (168.2) | 17.5 (166.6) | >0.99 |
| Albumin infused (ml); mean (SD) | 0 | 40.0 (127.5) | 39.8 (127.3) | 40.4 (128.0) | >0.99 |
| Isolyte infused; mean (SD) | 0 | 324.5 (710.1) | 324.4 (708.6) | 325.0 (716.3) | >0.99 |
| Epidural; n (%) | 0 | 235 (0.4) | 187 (0.4) | 48 (0.4) | >0.99^C^ |
| Epinephrine Infusion; n (%) | 0 | 96 (0.2) | 77 (0.2) | 19 (0.2) | >0.99^C^ |
| Vasopressin Infusion; n (%) | 0 | 33 (0.1) | 24 (0.1) | 9 (0.1) | >0.99^C^ |
| Milrinone Infusion; n (%) | 0 | 7 (0.0) | 6 (0.0) | 1 (0.0) | >0.99^F^ |
| Dobutamine Infusion; n (%) | 0 | 21 (0.0) | 18 (0.0) | 3 (0.0) | >0.99^F^ |
| Dopamine Infusion; n (%) | 0 | 264 (0.4) | 211 (0.4) | 53 (0.4) | >0.99^C^ |
| Norepinephrine Infusion; n (%) | 0 | 91 (0.2) | 79 (0.2) | 12 (0.1) | >0.99^C^ |
| Phenylephrine Infusion; n (%) | 0 | 15703 (26.6) | 12512 (26.5) | 3191 (27.1) | >0.99^C^ |
| Blood Products Total (ml); mean (SD) | 0 | 24.4 (153.8) | 24.2 (150.3) | 25.4 (167.2) | >0.99 |
| Crystalloids Total (ml); mean (SD) | 0 | 1496.1 (1127.0) | 1494.0 (1122.8) | 1504.7 (1143.8) | >0.99 |
| CVL present; n (%) | 0 | 1174 (2.0) | 926 (2.0) | 248 (2.1) | >0.99^C^ |
| Arterial Line present; n (%) | 0 | 4940 (8.4) | 3909 (8.3) | 1031 (8.7) | >0.99^C^ |
| Bicarbonate (preop); mean (SD) | 17556 | 25.9 (3.6) | 25.9 (3.6) | 26.0 (3.6) | >0.99 |
| Hemoglobin (preop); mean (SD) | 11877 | 11.5 (2.4) | 11.5 (2.4) | 11.5 (2.4) | >0.99 |
| Platelet Level (preop); mean (SD) | 11763 | 258.9 (119.8) | 259.3 (121.0) | 257.4 (114.7) | >0.99 |
| Last PACU Temp; mean (SD) | 71 | 97.9 (1.0) | 97.9 (1.1) | 97.9 (0.8) | >0.99 |
| PACU Urine (mL); mean (SD) | 54846 | 325.7 (304.2) | 325.6 (307.9) | 326.2 (289.8) | >0.99 |
| Non-Blood Fluids (ml), mean (SD) | 0 | 1536.1 (1190.6) | 1533.9 (1186.0) | 1545.1 (1209.0) | >0.99 |
| Room Air at discharge; n (%) | 0 | 22457 (38.1) | 17965 (38.1) | 4492 (38.1) | >0.99^C^ |
| Non-surgical anesthesia time; mean (SD) | 1132 | 60.5 (23.6) | 60.4 (23.7) | 60.8 (23.1) | >0.99 |
| Whole Blood transfused; (mean (SD) | 0 | <0.01 (<0.01) | <0.01 (<0.01) | <0.01 (<0.01) | >0.99 |
| D5 or D10 infused; mean (SD) | 0 | 1.7 (35.3) | 1.7 (36.1) | 1.4 (32.1) | >0.99 |
| Starches; n (%) | 0 | 1 (<0.01) | 1 (<0.01) | 0 (<0.01) | >0.99^F^ |
| Dextran; n(%) | 0 | 1 (<0.01) | 0 (<0.01) | 1 (<0.01) | >0.99^F^ |

^* Of note, a minimum or maximum PACU value (e. g. HR, SBP, RR< Aldrete) is measured in the final hour of the PACU stay. PACU: Post-Anesthesia Care Unit; HR: Heart Rate; SBP: Systolic Blood Pressure; MAP: Mean Arterial Pressure; RR: Respiratory Rate; EBL: Estimated Blood Loss; pRBC: packed Red Blood Cells; FFP: Fresh Frozen Plasma; CVL: Central Venous Line. In the p-value column, “C” denotes Chi-squared test and “F” denotes Fisher-exact test.^

**Supplemental Figure Legends.**

**Supplemental Fig. 1 Scorecard bins and similarities and differences of training and test data.** Bar graphs indicate the percentage of the training (gray) and test (black) population for each of the five scoring bins (denoted by percentage on *left* y-axis). The lines and indicate the percentage of UCE that occurs within each bin category for training (gray) and test (black) datasets (denoted by percentage on *right* y-axis). * indicates a significant difference in event rate of UCE within that particular bin

**Supplemental Fig. 2 All Shapley values that affect patient UCE risk.** On the left, the 10 most influential variables for patients whose estimated probability of UCE was greater than 20% are listed. Each patient is visualized as a path moving from lower risk (left-hand side) to higher risk (right-hand side) as variables of increasing importance (moving from bottom to top) are evaluated for that patient. The greater a patient moves to the right, the more risk that variable provided for that patient. If they move to the left, the variable caused a decrease in risk of UCE for that patient. Of note, any value labeled as “Maximum PACU” (e. g. HR, RR, Aldrete, SBP) is measured in the last hour of the PACU stay


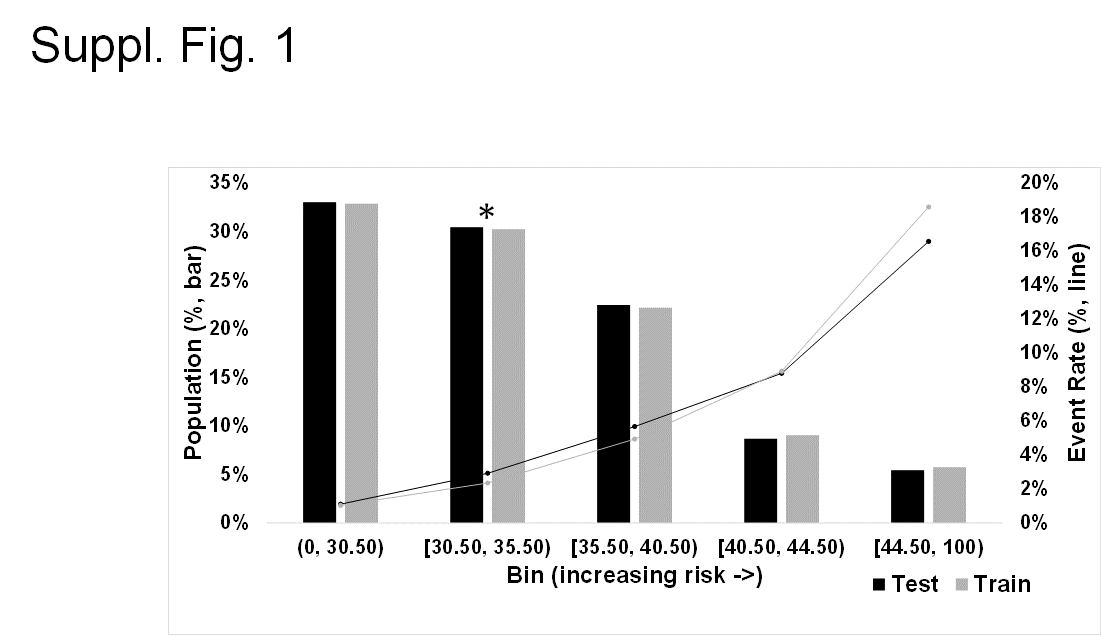


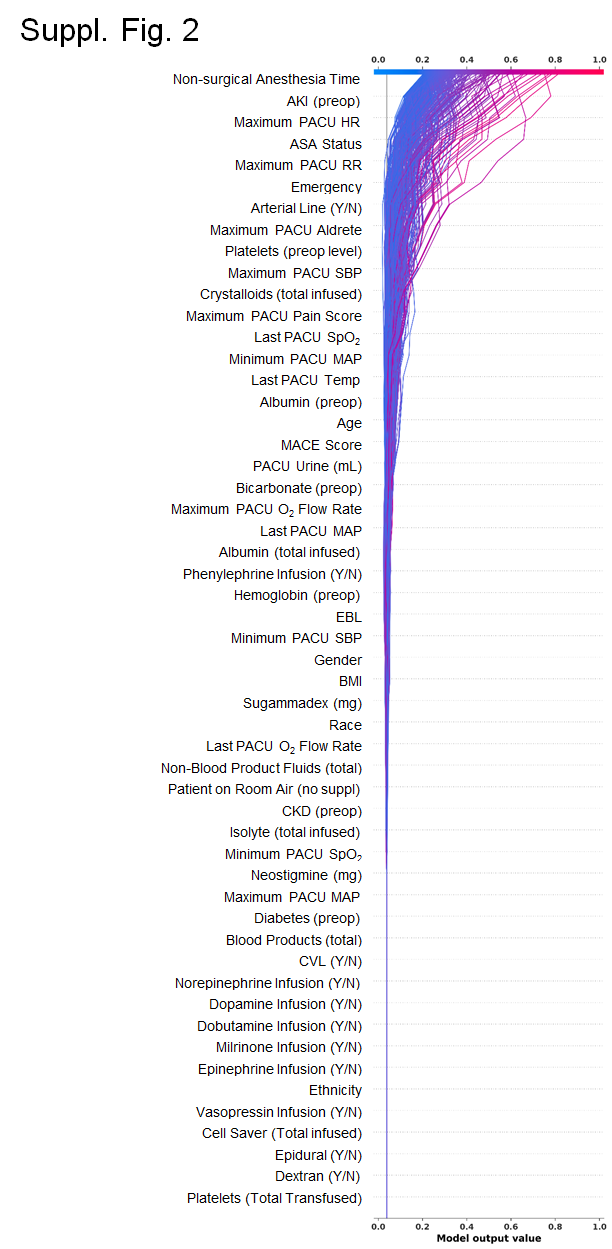

Supplement: Supplementary file 1 — Supplementary Material 1 [file 10916_2024_2085_MOESM1_ESM.docx]
